# Auxin Metabolite Balance During Haploid and Zygotic Oat Embryo Development—Quantitative and Localization Studies

**DOI:** 10.3390/ijms26125737

**Published:** 2025-06-15

**Authors:** Kinga Dziurka, Michał Dziurka, Marzena Sujkowska-Rybkowska, Kamila Laskoś, Magdalena Grela, Ewa Muszyńska

**Affiliations:** 1 The Franciszek Górski Institute of Plant Physiology, Polish Academy of Sciences, Niezapominajek 21, 30-239 Kraków, Poland; michal.dziurka@gmail.com (M.D.); k.laskos@ifr-pan.edu.pl (K.L.); m.grela@ifr-pan.edu.pl (M.G.); 2 Department of Botany, Institute of Biology, Warsaw University of Life Sciences-SGGW, Nowoursynowska 159, Building 37, 02-776 Warsaw, Poland; marzena_sujkowska@sggw.edu.pl (M.S.-R.); ewa_muszynska@sggw.edu.pl (E.M.)

**Keywords:** *Avena sativa*, embryogenesis, IAA amino acid conjugates, IAA immunolocalization, phytohormones

## Abstract

Auxins play a critical role in establishing the embryo axis and embryonic pattern. Our study aimed to determine the developmental stage of 21-day old oat (*Avena sativa* L.) haploid embryos, obtained by distant crossing with maize, and examined oat zygotic embryos at different developmental stages for their levels of endogenous indole-3-acetic acid (IAA), its metabolites, and IAA localization. The content of auxin metabolites was determined by HPLC-MS/MS, while IAA visualization in embryos was performed by immunohistochemistry and observed under confocal microscopy. We found that 21-day-old haploid embryos contained half the IAA concentration of age-matched zygotic embryos. Simultaneously, the total conjugated auxins (IAA-Asp, IAA-Glu, meIAA) were higher than in zygotic embryos, regardless of their age. Immunolocalization revealed IAA accumulation in embryos aligned with regions of tissue differentiation (e.g., shoot apical meristem, radicle primordium, and coleptile). We conclude that limited morphogenetic progression, evidenced by microscopic sections accompanied by changes in IAA content and distribution in haploid embryos, indicates a developmental stage earlier than the coleoptilar stage of zygotic embryos which occurs 9 days after pollination. Our findings may be useful in embryo rescue techniques, suggesting modulation of auxin concentration in in vitro culture.

## 1. Introduction

Phytohormones are a group of organic compounds responsible for regulating all stages of plant ontogeny and responding to environmental stimuli. Acting as signalling molecules, they regulate gene expression and communication between different signalling pathways [1]. Among them are auxins, a group of compounds with a similar chemical structure, most notably indole-3-acetic acid (IAA). They are known primarily for regulating cell elongation, plant movement, root formation, and fruit ripening [2]. Auxins are also important in early embryogenesis, coordinating the formation of the apical–basal axis of the embryo and the correct embryonic pattern [3,4,5].

The auxin metabolism in the plant cell, i.e., biosynthesis, conjugate formation and degradation, is well-characterized [6]. IAA in the cell may be produced by the oxidation of indole-3-butyric acid (IBA), which takes place in the peroxisome, or by biosynthesis from L-tryptophan (Trp) produced in the plastid [6]. The site of Trp-dependent IAA biosynthesis is the cytosol. Among Trp-dependent IAA synthesis pathways in *Arabidopsis*, the only fully understood pathway is the two-step indole-3-pyruvic acid (IPyA) mediated pathway. In the first step, Trp is converted by TAA1/TAR family aminotransferases to IPyA, and in the second step, IPyA is decarboxylated by YUCCA family enzymes to IAA [7]. IAA is reversibly inactivated by forming amide (e.g., IAA-Ala, IAA-Leu, IAA-Phe) and ester conjugates (IAA-glc, meIAA) or irreversibly by direct oxidation to 2-oxindole-3-acetic acid (oxIAA), which occurs in the cytoplasm [8,9].

IAA moves between cells either by diffusion in a protonated form or via the AUX1/LAX, PGP, and PINs transporters in the cell membrane [10]. Intracellular transport of IAA between the cytoplasm and the endoplasmic reticulum or/and the cell nucleus is possibly mediated by non-canonical PINs and PILs transporters [11], while transport between cytoplasm and vacuole may occur via the WAT1 transporter [12]. The auxin response pathway involves the SCFTIR1/AFB-Aux/IAA receptor, which acts in the cell nucleus. It consists of two co-receptor proteins, TIR1/AFB and Aux/IAA [13]. Under conditions of low auxin concentration, the Aux/IAA protein binds to the ARF transcription factor, inhibiting the transcription of auxin-induced genes. If the auxin concentration is high, the hormone molecules are bound into the SCFTIR1/AFB-Aux/IAA complex, Aux/IAA proteins are degraded, and the released ARF allows the transcription of auxin-induced genes [14]. ARF proteins recognize the core TGTC motif (Auxin Responsive Elements, Aux-Re) in the promoters of auxin-regulated genes [15]. ARF transcription factors regulate the expression of genes from the Aux/IAA, SAUR, and GH3 families [1].

A very good compendium of knowledge on the role of auxins and their transporters in the formation of the embryonic pattern was provided by Capron et al. [16], who described in detail the molecular mechanism responsible for this process in the model dicotyledonous plant—*Arabidopsis*. Maize and rice are key model monocots for molecular embryogenesis studies [17,18], whereas research on embryo development in other cereals is not as advanced [19]. This is especially true for haploid embryos, which are the prerequisite step necessary for the production of doubled haploids with exceptional genetic values [20]. They are eagerly used in breeding programs because they significantly accelerate the creation of new cultivars [20]. Currently, breeding is faced with the challenge of producing cultivars that can cope with the ubiquitous water shortage, are resistant to diseases, and, at the same time, produce high and stable yields [21]. Oat (*Avena sativa*, Poaceae) is of special interest among many cereals due to its exceptional health-promoting properties [22]. Oat is considered recalcitrant to haploidization [23], therefore the development of a method for obtaining doubled oat haploids was a milestone in the breeding of this species. Although there are practical protocols for obtaining haploids and doubled haploids of oat by distant crossing with maize [24,25,26], the mechanism of haploid embryo formation remains unclear. Besides this, the role of auxins in the development of haploid oat embryos has remained obscure. It was shown that ovaries in which an oat haploid embryo was formed as a result of distant crossbreeding with maize accumulated more IAA compared to ovaries in which the embryo did not develop [27]. In turn, morphological and anatomical observations of haploid oat embryos indicated developmental anomalies that may arise from the distribution of auxins [28] as these phytohormones are responsible for polarity and the formation of the proper embryonic pattern [5]. Additionally, the IAA level in haploid oat embryos isolated at the optimal age, allowing for their further development (i.e., 21 days after pollination with maize pollen), was several times lower than in zygotic embryos of the same age [28]. The less advanced structure of haploid embryos, combined with low IAA and high IBA concentration, suggests their developmental delay compared to zygotic embryos [28].

In this study, we made an effort to estimate the maturation level of 21-day-old haploid oat embryos of the ‘Akt’ cultivar by analysing the profile of auxin metabolites and the subcellular IAA localization in the background of zygotic embryos at different stages of development. We hypothesized that haploid embryos exhibit auxin profiles distinct from counterparts, contributing to developmental arrest.

## 2. Results

### 2.1. Auxin Metabolites During Oat Embryo Development

The content of the auxin precursor IBA was the highest (0.74 nmol/g DW) (Table 1) at 1 DAP, i.e., at the beginning of embryogenesis, and then decreased in the following days, reaching its minimum in embryos 14 and 21 DAP (0.10 and 0.05 nmol/g DW, respectively). The accumulation of IAA increased as the zygotic embryos developed, reaching maximum values at 9 and 14 DAP (5.97 and 5.30 nmol/g DW, respectively) and then rapidly decreased. The meIAA remained low (0.10–0.2 nmol/g DW) across zygotic embryo stages. The concentration of auxin amino acid conjugate IAA-Asp showed an increasing trend on subsequent measurement days, reaching the highest value at 21 DAP (1.26 nmol/g DW). At the same time, IAA-Glu had similar values except at 12 DAP, when it was higher. In turn, oxIAA content was significantly similar (*p* < 0.05) during the entire embryo development, except for 9 DAP, when a higher concentration (0.6 nmol/g DW) was recorded than on the other days (when it ranged from 0.12 to 0.34 nmol/g DW).

**Figure 1:**
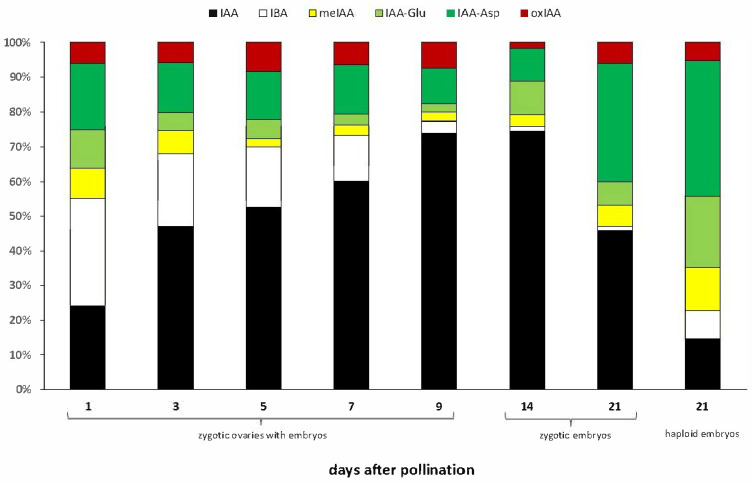
The percentage content of individual compounds in the total pool of the auxin metabolites (treated as 100%) in ovaries with zygotic embryos and both haploid and zygotic embryos of oat.

Shifting ratios of individual auxin metabolites were observed as zygotic embryos developed. At 1 and 3 DAP, precursor (IBA) and inactivated forms (IAA-Asp) predominated in the pool of analysed auxin metabolites, mainly IBA (31% and 21%, respectively) and the IAA-Asp amino acid conjugate (19% and 15%, respectively) (Figure 1). In the following days after pollination, as the content of active IAA increased, the proportion of inactive forms in the auxin pool decreased. In particular, in 14 and 21 DAP zygotic embryos, the IBA precursor constituted only 1% of all auxin metabolites. At 21 DAP, we again observed a reversal of the proportions of IAA and other auxin metabolites in favour of the inactivated forms, as was the case in the first days after pollination (Figure 1). The predominant form of inactivated auxins was the IAA-Asp amino acid conjugate.

The 21-day haploid embryos contained half the amount of IAA compared to the 21-day zygotic embryos. At the same time, the IAA content in haploid embryos remained at the same level as in the 1–7 DAP and 21 DAP zygotic embryos (Table 1). The content of the auxin precursor IBA in haploid embryos was at a similar level to that in zygotic embryos starting from 3 DAP (Table 1). At the same time, a substantial accumulation of inactive forms of auxin meIAA, IAA-Glu, and IAA-Asp was observed in 21-day-old haploid embryos (Figure 1). They contained a few times more of these metabolites than zygotic embryos of the same age (Table 1). Interestingly, the proportion between IAA and inactivated forms of auxin in haploid embryos resembles those at the beginning of embryogenesis of zygotic embryos, with 15% IAA and 85% of precursor and other auxin metabolites, of which the dominant ones turned out to be IAA-Asp and IAA-Glu amino acid conjugates (39% and 21%, respectively), which in turn makes them similar to 21-day zygotic embryos.

### 2.2. Distribution of IAA During Oat Embryo Development

**Figure 2:**
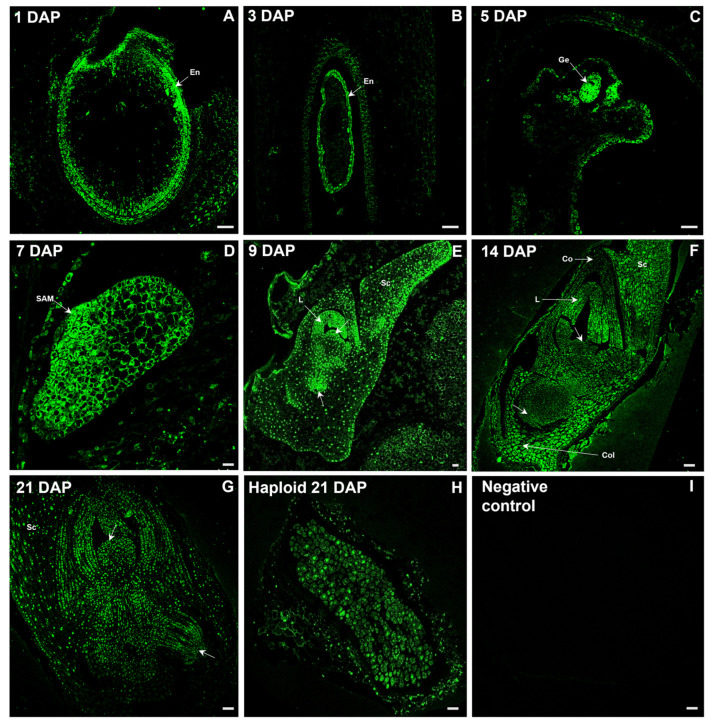
Confocal images of IAA immunolocalization at different stages of zygotic embryo development and in haploid embryos of oat. (**A**) An ovary at 1 DAP with a visible IAA signal (green) in the forming endosperm. (**B**) An ovary at 3 DAP with a visible green signal in the endosperm (En). (**C**) An ovary at 5 DAP with an intense green signal of the globular proembryo (Ge). (**D**) Higher magnification of the embryo in the coleoptilar stage with an intense IAA signal in the forming shoot apical meristem (SAM). (**E**) The oat embryo at 9 DAP showing a strong IAA signal in both meristems (arrows) and the dorsal–apical part of the scutellum (Sc). (**F**) The embryo at 14 DAP with visible strong signal in the coleorhiza (Col) and a less intense signal in the meristems (arrows), juvenile leaves (L), coleoptile (Co), and scutellum (Sc). (**G**) The embryo at 21 DAP with visible weak IAA signal in embryo tissues. (**H**) The haploid 21 DAP embryo with weak IAA signal. (**I**) Negative control without primary antibody—no fluorescent signal is detected. Arrow—meristem; Co—coleoptile; Col—coleorhiza; Ge—globular proembryo; L—leaf; SAM—shoot apical meristem; Sc—scutellum. Polyclonal rabbit anti-IAA primary antibody (1:100) plus goat anti-rabbit IgG Alexa Fluor 488 conjugate as secondary antibody (1:100) (shown in green colour) were used. Scale bar = 50 µm, except for 3 DAP (**B**), where it is 10 µm.

Since auxins act as signalling molecules controlling embryogenesis, the temporal and spatial regulation of IAA concentration, and the occurrence of precursors and inactive forms in tissues are crucial for the establishment of the correct embryonic pattern. Figure 2 shows the developmental course of zygotic embryos, with particular attention to the temporal changes in the localization of free IAA. The fluorescent signal of IAA (green) was first noticed in the developing endosperm (Figure 2A,B), while in the proembryo it occurred in its multicellular globular stage observed 5 DAP (Figure 2C). At 7 DAP, the first morphogenetic events were identified, including the formation of shoot apical meristem (SAM) and radicle primordium at opposite positions, and the coleoptile primordium located in close relation to the SAM. At this so-called coleoptilar stage, the most intensive IAA signal was shown in the mentioned structures, which determined the embryo axis, while the auxin signal from the chalazal cells of differentiating scutellum was strongly reduced (Figure 2D). Then, the immunofluorescence signal was easily noticeable in both meristems, the first developed leaf, the protrusion of the second leaf, and the dorsal–apical part of the scutellum as revealed in 9 DAP (Figure 2E). In this time, the oat embryo seemed to be morphologically completed and reached the stage L1. Subsequently, maturation processes, together with changes in the intensity and location of the auxin signal, took place. At 14 DAP, fluorescence occurred in the specialized tissue called coleorhiza, which covers the radicle (Figure 2F). Other embryo regions showed progressive weakening of IAA signals in the embryo axis, juvenile leaves, coleoptile, and scutellum (Figure 2F). This signal attenuation continued until, by 21 DAP, distinct areas of elevated free auxin accumulation became indistinguishable (Figure 2G). The haploid embryo was structurally less advanced than the zygotic embryo of the same age. At 21 DAP, the haploid embryo showed no observable morphogenetic activity, while only weak IAA fluorescence was detected throughout the embryo (Figure 2G).

**Figure 3:**
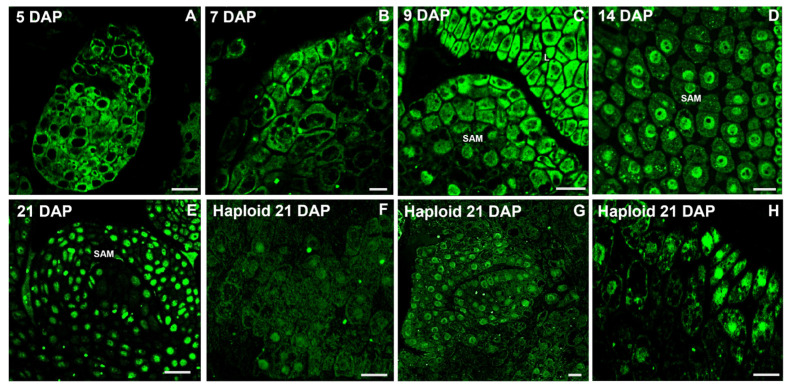
Close-up on IAA distribution in meristematic cells of developing shoot apical meristems (SAM) at various stages of zygotic and haploid oat embryos. (**A**) The globular proembryo at 5 DAP with an intensive green IAA signal in the cell cytoplasm on the entire embryo surface. (**B**) Higher magnification of the embryo in the coleoptilar stage with a forming SAM, the cells of which exhibited an intensive IAA signal in the cytoplasm. (**C**) The embryo at 9 DAP with a strong IAA signal in the cytoplasm of the shoot apical meristem (SAM) and juvenile leaves (L). (**D**) Change in IAA localization in SAM cells of the embryo at 14 DAP to signal detectable only in the cell nuclei. (**E**) Apical meristem of the embryo at 21 DAP with IAA signal in the cell nuclei. (**F**–**H**) Different zones of the haploid 21 DAP embryos with a weak signal in both the cytoplasm and nuclei. Polyclonal rabbit anti-IAA primary antibody (1:100) plus goat anti-rabbit IgG Alexa Fluor 488 conjugate as secondary antibody (1:100) (shown in green colour) were used for IAA immunolocalization. Scale bar = 10 µm.

Results of a deeper insight into IAA localization in particular cells of meristematic zones of developing shoot apical meristems during the course of embryogenesis are shown in Figure 3. Higher magnifications of various zygotic embryo stages revealed an intense IAA signal only in the cytoplasm of the proliferating cells of proembryos at 5 and 7 DAP, while 9 DAP auxin was detected additionally in the nuclear region. Later, IAA localization shifted markedly, and in 14- and 21-day-old embryos, IAA was visualized mainly in the nucleus and almost not at all in the cytoplasm. In turn, the IAA signal in cells of haploid embryos was not only weaker compared to zygotic embryos, but also distributed both in the cytoplasm and nucleus.

## 3. Discussion

Knowledge about the biosynthesis, metabolism and molecular mechanism of auxin action is based mainly on studies of the model plant *Arabidopsis thaliana*. The current challenge facing scientists is to transfer the knowledge acquired from model plants to crop species. The sites and pathways of de novo auxin biosynthesis are still insufficiently understood [29]. The two basic methods for visualizing auxins are the use of auxin-responsive promoter elements, such as DR5-type reporters [30], and the use of antibodies targeted against IAA. The first indirect method has primarily localized IAA in embryos in the dicotyledonous plant *Arabidopsis* [31]. Among monocotyledonous plants, the localization of IAA using the direct immunohistochemical method in maize embryos was demonstrated by Forestan et al. [32]. In the globular stage of the maize embryo, a strong free IAA signal was visualized at the top of the embryo and a very weak one in the suspensor. Then, in the coleoptilar stage of maize embryogenesis, the authors observed a gradient of IAA distribution starting from a high signal at the top of the scutellum and decreasing towards the suspensor. We believe that the described maize embryo may correspond to a 7 DAP oat embryo with auxin accumulation in the region of the developing coleoptile and SAM. The similarity between maize and oat embryos can also be observed at the morphogenesis stage, which is represented by the 9 DAP embryos in the case of oat. IAA accumulation in oat embryos was visible at the tip of the leaf primordia, and in the corpus of the SAM and RAM (root apical meristem). In both maize and oat embryos, IAA accumulation coincided with sites of differentiation and organ establishment. Against the background of typical embryogenesis of oat zygotic embryos, haploids showed a significant delay in development, lack of visible tissue differentiation and clear sites of IAA accumulation, which leads us to conclude that these embryos most closely correspond to the proembryonic stage of zygotic embryos. The mechanism of reduced growth rate can be explained by conjugate buildup, especially if accompanied by a decrease in free IAA, which may suppress IAA signalling, thereby stalling morphogenesis [14]. However, based on our previous observations, it should be mentioned that haploid oat 21 DAP embryos may also achieve slightly more advanced stages [24], but their growth is still retarded by about 10–14 days compared to zygotic embryos [14].

The content of active auxin IAA increases during the development of the oat ‘Akt’ zygotic embryo until 9 DAP and remains at a similar level at 14 DAP, after which it decreases. We have previously observed the same trend for the oat ‘Krezus’ cultivar [24]. For comparison, the highest IAA content in wheat kernels was recorded at 18 DAP and declined by 25 DAP [33]. At the beginning of the zygotic development of an oat embryo, IAA was mainly localized in the cytoplasm. At 9 DAP, an additional IAA fluorescence signal was observed in the cell nucleus, while at 14 and 21 DAP, it was observed mainly in the cell nucleus. Considering that IAA biosynthesis occurs in the cytoplasm [6], it can be assumed that intensive auxin production continues until 9 DAP. Since the 9 DAP embryo appears to be morphologically fully formed, the following days must have been devoted to embryo growth and maturation. This likely reflects auxin-dependent gene regulation, and thus, a strong IAA fluorescence signal was observed in the cell nucleus of 14 and 21 DAP embryos. A stable coreceptor complex TIR1/AFB-auxin-Aux/IAA is formed at higher auxin concentrations in the nucleus. Aux/IAA is ubiquitinated and then degraded in the proteasome. As a result, ARF proteins are released from repression and can activate transcription in target genes [34]. The decrease in IAA content in the pool of measured auxin metabolites at 21 DAP was accompanied by an increase in the percentage of inactive forms of auxin conjugates, mainly IAA-Asp. It can be assumed that some of the IAA was inactivated by binding to conjugates. This process, similar to IAA synthesis, occurs in the cell’s cytoplasm [6]. This may explain the weakening IAA signal in the cytoplasm of the 21 DAP embryo, as the anti-IAA antibody used in the experiment binds to free IAA and does not detect auxin conjugates. Similarly, the weak IAA signal in the cytoplasm, stronger in some cells of the haploid embryo nucleus, is associated with a low free IAA content (0.83 nmol/g DW) and a higher content of sum conjugates of 4.08 nmol/g DW.

Although the cytosol is considered the site of auxin conjugate formation, IAA-Asp, IAA-Glu, and oxIAA have been found in the cell nucleus [35], which indicates the possibility of their transport, probably a polar one, similar to free IAA. On the other hand, Porco et al. [36] claim that oxIAA is not transported, which raises the question of IAA oxidation in the cell nucleus. However, it is not known what role auxin conjugates play in the cell nucleus. IAA-Asp and IAA-Glu, in contrast to other IAA-amino acid conjugates, have long been considered irreversible auxin catabolites that cannot be hydrolyzed to free IAA in the plant [6,37]. Nevertheless, hydrolysis may reactivate these conjugates [38,39,40]. Also, Hayashi et al. [9] proposed a new model for the IAA inactivation pathway in which IAA-Asp and IAA-Glu are the storage forms of IAA, converted back to free IAA by ILR1/ILL aminohydrolases. In parallel, IAA-Asp and IAA-Glu conjugates can be irreversibly oxidized by DAO1 dioxygenase to oxIAA-Asp and oxIAA-Glu and further hydrolyzed by ILR1-like hydrolases to oxIAA, an inactive form of IAA [9]. The accumulation of IAA conjugates in the mature embryo and the possibility of their remobilization after a dormant period seem to make sense, as active auxins play a key role in the initial phase of seed germination [41]. In this context, the phenomenon of accumulation of auxin conjugates in haploid embryos, in which more advanced morphogenetic events and higher IAA concentrations would be expected at this age than observed until now, is intriguing and requires further investigation. At the same time, the fact of low free IAA content in haploid embryos carries a practical conclusion about the possibility of increasing the auxin content in the media used for the conversion of such embryos in in vitro cultures.

## 4. Materials and Methods

### 4.1. Obtaining Oat Embryos

Oat cv. ‘Akt’ (Strzelce Plant Breeding Ltd. IHAR Group, Strzelce, Poland) was sown in pots with a mixture of universal garden soil and sand in a 3:1 ratio. The plants were grown in a greenhouse, under natural light conditions, as previously described by Warchoł et al. [42]. During the oat flowering phase, single oat flowers were observed daily, marking the faded ones (with wilted anthers) as being 1 day after pollination (DAP). Ovaries or caryopses were collected on 1, 3, 5, 7, 9, 14 and 21 DAP. Samples 1–9 DAP constituted the ovary with the developing embryo. Samples 14 and 21 DAP were isolated embryos. Haploid embryos were obtained by cross-pollination of the oat ‘Akt’ cultivar and the maize ‘Waza’ cultivar. Flowers were emasculated pre-anthesis using tweezers, then two days later manually pollinated with maize pollen and sprayed with a 2,4-dichlorophenoxyacetic acid (2,4-D) solution (100 mg/L) 24 h later. Haploid embryos were excised from enlarged ovaries at 21 DAP. For details on obtaining haploid embryos, please refer to the protocol published by Skrzypek et al. [26].

### 4.2. Quantitation of Auxins by HPLC-MS/MS

The collected samples were frozen, then lyophilized, weighed (3 mg DW) and ground in a ball mill (MM 400, Retsch, Kroll, Germany) as previously described by Dziurka et al. [28]. The samples were spiked with [2H5]indole-3-acetic acid methyl ester (MeIAA-D5) and [2H5]indole-3-acetic acid (IAA-D5). These samples were then extracted using 1 mL of methanol/water/formic acid mixture (MeOH/H_2_O/HCOOH 15/4/1 *v*/*v*/*v*) and shaken for 5 min. A double extraction was performed, and the combined extracts were evaporated under a stream of nitrogen and reconstituted in 1 M HCOOH/5% MeOH for SPE loading. After purification on SPE cartridges (BondElutPlexa PCX, 30 mg, 1 mm, Agilent, Santa Clara, CA, USA), the fraction with auxins was evaporated again, reconstituted in 70 μL of acetonitrile (ACN), filtered (0.22 μm nylon membrane), and analysed using an HPLC system equipped with UHPLC (Agilent Infinity 1260, Agilent, Waldbronn, Germany) and a triple quadrupole mass spectrometer (Agilent 6410, Agilent, USA) with electrospray ionization (ESI). An AscentisExpres RP-Amide analytical column (2.7 μm, 2.1 mm × 150 mm; Supelco, Bellefonte, PA, USA) and a linear gradient of water vs. ACN both with 0.01% HCOOH was used (0.5 mL/min, from 3% to 46% ACN in 24 min, at 60 °C). Indole-3-butyric acid (IBA), indole-3-acetic acid methyl ester (meIAA), indole-3-acetic acid (IAA), indole-3-acetylglutamic acid (IAA-Glu), indole-3-acetyl-aspartic acid (IAA-Asp), and oxoindole-3-acetic acid (oxIAA) were determined. Multiple reaction transition monitoring (MRM), reported by Dziurka et al. [28], was used to identify and quantify all compounds of interest. Standards were from OlChemim (Olomouc, Czech Republic), and solvents from Sigma-Aldrich (Poznań, Poland).

### 4.3. Immunohistochemical Localization of IAA by Confocal Laser Microscopy

Samples for histological analysis were taken 1, 3, 5, 7, 9, 14 and 21 DAP and fixed in 4% paraformaldehyde in Microtubule Stabilization Buffer (MSB) for 2 h and then washed four times with MSB. After dehydration through an ethanol gradient with the addition of dithiothreitol (DTT), the samples were embedded in butyl-methyl-methacrylate (BMM) resin. Fixation and embedding parameters were as previously described by Sujkowska-Rybkowska et al. [43]. Semi-thin sections (2 μm thick) were cut using a Reichert Jung microtome (Leica, Wetzlar, Germany) and placed on Silane-Prep slides (Sigma-Aldrich) in a drop of distilled water. After removing the BMM resin using acetone, the slides were immersed in blocking solution [5% bovine serum albumin (BSA) (Sigma-Aldrich) in 0.01 M phosphate-buffered saline (PBS)] for 30 min at RT. Slides were rinsed in 0.01 M PBS buffer with 0.05% Tween 20 four times, then once more in 0.01 M PBS. The primary polyclonal rabbit anti-IAA antibody (AS09 445, Agrisera, Vännäs, Sweden) was diluted 1:100 in PBS containing 0.5% (*w*/*v*) BSA and incubated overnight at 4 °C. Next, the slides were washed with PBS-Tween and PBS, the same as above. To visualize antibodies bound to IAA, the sections were treated with secondary goat anti-rabbit antibody IgG with attached Alexa Fluor 488 (Thermo Fisher Scientific, Waltham, MA, USA) (1:100) in PBS with 0.5% BSA for 1.5 h at RT in the dark. After incubation, the sections were washed with PBS-Tween and PBS and imaged by confocal laser scanning microscope (Leica TCS Sp5; argon laser; Ex/Em range of 485–570 nm). In the present experiments, the specificity of immunolocalization was confirmed by incubating the sections without primary antibody (negative control). Three biological replicates, each with 10 different embryos, were labelled.

### 4.4. Statistics

Means ± standard errors (SE) were obtained for three replicates. One-way analysis of variance (ANOVA) was applied to estimate the effect of embryo type (haploid/zygotic) and developmental stage on IAA and its metabolites. Post hoc Duncan’s multiple range test was used for means separation (*p* < 0.05). Data were analysed with the STATISTICA 10.0 (Stat-Soft, Inc., Tulsa, OK, USA) software package.

## 5. Conclusions

The present experiment allows for assessment of physiological maturity and the developmental potential of haploid oat embryos isolated 21 days after cross-pollination with maize. We have shown that the haploid embryos were clearly delayed compared to the zygotic ones. The lack of visible meristem establishment suggests a developmental phase corresponding to stages of zygotic embryos earlier than 9 DAP. It was also confirmed by cellular IAA localization, which in haploid cells was similar to that observed in 9 DAP zygotic embryos, as the fluorescence signal originated from both the nucleus and cytoplasm, although it was significantly weaker. These findings highlight that auxin level and distribution are strongly correlated with morphogenetic processes in oat embryos, while comparing them between zygotic and haploid ones provides information about those embryos’ developmental equivalence. Thus, our results suggest that auxin modulation, the demand for which changes during embryogenesis, could improve haploid embryo rescue protocols under in vitro conditions.

## Figures and Tables

**Table 1:** The content of auxin metabolites in zygotic ovaries with embryos, zygotic embryos, and haploid embryos of oat after 1, 3, 5, 7, 9, 14 and 21 days after pollination.

Metabolite [nmol/g DW]	Zygotic Ovaries with Embryos					Zygotic Embryos		Haploid Embryos
	1 DAP	3 DAP	5 DAP	7 DAP	9 DAP	14 DAP	21 DAP	21 DAP
IAA	0.57 ± 0.5 b *	1.43 ± 0.08 b	2.09 ± 0.51 b	2.77 ± 0.13 b	5.97 ± 0.67 a	5.30 ± 1.47 a	1.70 ± 0.52 b	0.83 ± 0.02 b
IBA	0.74 ± 0.22 a	0.63 ± 0.25 bc	0.69 ± 0.04 bc	0.61 ± 0.06 bc	0.28 ± 0.03 cd	0.10 ± 0.01 d	0.05 ± 0.01 d	0.46 ± 0.12 bcd
meIAA	0.21 ± 0.03 bc	0.20 ± 0.02 bc	0.10 ± 0.02 c	0.14 ± 0.00 bc	0.21 ± 0.01 bc	0.22 ± 0.04 b	0.23 ± 0.06 b	0.71 ± 0.04 a
IAA-Asp	0.45 ± 0.11 c	0.44 ± 0.29 c	0.55 ± 0.20 c	0.65 ± 0.06 bc	0.83 ± 0.09 bc	0.68 ± 0.12 bc	1.26 ± 0.10 b	2.21 ± 0.65 a
IAA-Glu	0.26 ± 0.09 c	0.15 ± 0.01 c	0.21 ± 0.01 c	0.14 ± 0.01 c	0.19 ± 0.01 c	0.69 ± 0.06 b	0.25 ± 0.03 c	1.16 ± 0.14 a
oxIAA	0.14 ± 0.04 b	0.18 ± 0.08 b	0.34 ± 0.12 b	0.30 ± 0.05 b	0.60 ± 0.06 a	0.12 ± 0.02 b	0.22 ± 0.05 b	0.30 ± 0.04 b
Sum of conjugates	0.93 c	0.79 c	0.86 c	0.93 c	1.23 bc	1.60 b	1.74 b	4.08 a

* Values are means of three replicates ± standard error. Means indicated by the same letter do not significantly differ at *p* < 0.05 according to one-way ANOVA and post hoc Duncan’s test. IAA—indole-3-acetic acid, IBA—indole butyric acid, meIAA—indole-3-acetic acid methyl ester, IAA-Glu—indole-3-acetyl-glutamic acid, IAA-Asp—indole-3-acetyl-aspartic acid, oxIAA—oxoindole-3-acetic acid, DAP—days after pollination.

## Data Availability

All data are contained within the article. The datasets used and analysed during the current study are available from the corresponding author on reasonable request.
